# Ultrasound as a Promising Tool for the Green Extraction of Specialized Metabolites from Some Culinary Spices

**DOI:** 10.3390/molecules26071866

**Published:** 2021-03-25

**Authors:** Jana Šic Žlabur, Marko Brajer, Sandra Voća, Ante Galić, Sanja Radman, Suzana Rimac-Brnčić, Qiang Xia, Zhenzhou Zhu, Nabil Grimi, Francisco J. Barba, Nataša Hulak

**Affiliations:** 1 Faculty of Agriculture, University of Zagreb, Svetošimunska cesta 25, 10000 Zagreb, Croatia; jszlabur@agr.hr (J.Š.Ž.); mbrajer1@gmail.com (M.B.); svoca@agr.hr (S.V.); agalic@agr.hr (A.G.); sradman@agr.hr (S.R.); srimac@pbf.hr (S.R.-B.); nhulak@agr.hr (N.H.); 2 State Key Laboratory of Quality Research in Chinese Medicine, Institute of Chinese Medical Sciences, University of Macau, Macau 999078, China; xqiang0713@hotmail.com; 3 College of Food Science and Engineering, Wuhan Polytechnic University, Wuhan 430023, China; zhenzhouzhu@126.com; 4 ESCOM, EA 4297 TIMR, Centre de Recherch Royallieu, Université de Technologire de Compiègne, CS 60319, CEDEX, 60203 Compiègne, France; nabil.grimi@utc.fr; 5 Nutrition and Bromatology Area, Department of Preventive Medicine and Public Health, Food Science, Toxicology and Forensic Medicine, Faculty of Pharmacy, Universitat de València, Avda. Vicent Andrés Estellés, 46100 Burjassot, València, Spain

**Keywords:** bioactive compounds, antioxidant capacity, antimicrobial activity, ultrasonic extraction, spices

## Abstract

Spices are a popular food of plant origin, rich in various phytochemicals and recognized for their numerous properties. The aim of the study was to evaluate the antioxidant and antimicrobial activity, as well as the content of specialized metabolites, of aqueous extracts of three spice species––garlic (*Allium sativum* L.), ginger (*Zingiber officinalle* L.) and turmeric (*Curcuma longa* L.)––prepared by green extraction methods. Ultrasound treatment increased the chromaticity parameter b value of turmeric and ginger extracts, thus indicating a higher yellow color predominantly due to curcuminoids characteristic of these species. Ultrasound-assisted extraction significantly increased the content of total soluble solids, phenolic compounds, total carotenoids and vitamin C. The temperature of the system was also an important factor, with the highest (70 °C) conditions in ultrasound-assisted extraction having a positive effect on thermolabile compounds (vitamin C, phenolics, total carotenoids). For example, turmeric extract treated with ultrasound at 70 °C had up to a 67% higher vitamin C content and a 69.4% higher total carotenoid content compared to samples treated conventionally at the same temperature, while ginger extracts had up to 40% higher total phenols. All different concentrations of spice extracts were not sufficient for complete inhibition of pathogenic bacterial strains of *Salmonella*, *L. monocytogenes* and *S. aureus*; however, only garlic extracts had an effect on slowing down the growth and number of *L. monocytogenes* colonies. Spice extracts obtained by ultrasonic treatment contained a significantly higher level of bioactive compounds and antioxidant capacity, suggesting that the extracts obtained have significant nutritional potential and thus a significant possibility for phytotherapeutic uses.

## 1. Introduction

Spices have had wide application and significant use since ancient times, both as flavourings and as remedies in folk medicine because they are rich in valuable phytochemicals and thus have numerous health benefits. Bioactive compounds such as curcumin (from turmeric), gingerol (from ginger) or natural organosulfur compounds from garlic show potent anti-cancer and anti-diabetic activity; effectively lower total cholesterol, LDL cholesterol, serum glucose and triacylglycerols; and exhibit significant cardioprotective and anti-inflammatory properties [1]. One of the most well-known is their activity against bacteria, fungi, yeasts and microbial toxin synthesis as well as their antioxidant effects. Garlic, more specifically allicin from garlic, is considered to be one of the most powerful natural antibiotics [2], while phytochemicals (flavonoids, alkaloids, terpenes, saponins, glycosides, phenolic compounds) are among the most important natural bioactive compounds. Their main function is to detoxify reactive oxygen species (ROS), which are the direct consequence of unwanted oxidation in human cells [3].

Natural antimicrobials derived from plant organisms and intended for food processing should have bactericidal and fungicidal activity, be active at low concentrations, be heat and pH stable, and be free from toxicity and impurities [4]. The phytochemicals in spices could potentially interact with, and reduce, foodborne pathogens [5,6], which often exhibit multidrug resistance as has been increasingly demonstrated by bacteria like *Salmonella* sp., *Listeria monocytogenes*, and *Staphylococcus aureus* [7,8,9]. Because of their pathogenic multiplication in the host, the aforementioned bacterial species cause a variety of diseases that are not limited to gastrointestinal infections like salmonellosis but can also cause severe clinical problems due to their ability to produce listeriosis [10] or the toxin-mediated virulence of *S. aureus* [11]. Apart from the antibiotic resistance and ubiquity of this pathogen, it can also adapt and grow in a variety of physical conditions [12], which makes it extremely interesting to study.

Naturally occurring antioxidants are mainly polyphenolic compounds that often act as radical scavengers or chain-breaking antioxidants, chelators, quenchers, oxygen scavengers and regenerators that protect other antioxidants from radicalization [4]. Precisely because of their significant antimicrobial and antioxidant properties, spices have great potential to be used as natural food preservatives and have an extraordinary advantage over synthetic antioxidants and antimicrobials. Moreover, the antioxidant and antimicrobial activity of different spices differs depending on the matrix used, which correlates with the concentration and nature of the specific metabolites, mainly polyphenols, flavonoids, natural pigments and terpenes [13,14]. It is important to emphasize that combinations of individual polyphenols, medicinal herb species or food matrices, polyphenol-rich foods and polyphenols and other protective phytochemicals are key factors for influencing the bioactive properties of the final foods, and their synergistic effect is crucial for the prevention of associated chronic diseases [15].

In addition, spices have several processing possibilities and can be used fresh, dried, powdered, or as extracts [13]. The use of spice extracts of various bases is becoming more popular, with the usual conventional isolation techniques being increasingly replaced by modern non-invasive ones. Ultrasound-assisted extraction (UAE) is a promising method for the recovery of aromatic (volatile) and other phyto-ingredients from plant matrices, as well as for isolating various chemical compounds. The isolation of bioactive compounds from plant matrices is challenging, mainly because key factors causing faster mass transfer include high system temperatures, the use of organic solvents that are often harmful to health, and prolonged treatment time, which often leads to the degradation of sensitive (thermolabile) phytochemicals. UAE is considered one of the least invasive, highly energy-efficient and, most importantly, environmentally friendly methods, as evidenced by numerous studies. It is characterised by a fast and simple procedure that allows the extraction of a relatively large sample mass in a short time, while preserving the most sensitive phytochemicals such as antioxidants [16,17,18,19,20,21]. UAE uses ultrasonic waves of high intensity and low frequency to create transient cavitation, an effect that leads to the degradation of a cell’s walls and membrane, resulting in the release of soluble ingredients into the surrounding medium (solvent). Apart from extraction, ultrasound has been shown to have some effect on the inactivation of microorganisms, but with major limitations like insufficiently high temperatures [22]. Regarding ultrasonic sterilization, the most effective technique for reducing microorganisms is combining ultrasound with heat, a process known as thermosonication [23]. Therefore, the aim of the study was to evaluate the antioxidant and antimicrobial activity, as well as the content of specialized metabolites of aqueous extracts of spice species: garlic *(Allium sativum* L.), ginger (*Zingiber officinalle* L.) and turmeric (*Curcuma longa* L.) prepared by green extraction methods (conventional and ultrasound-assisted)

## 2. Results and Discussion

### 2.1. Physicochemical Properties of Spice Extracts

Physicochemical properties are one of the tools used to verify the quality of plant material. However, in this study, the analysis of the reported properties of the prepared aqueous spice extracts referred primarily to the quality and stability of the extracts themselves, regardless of the type of spice used. Indeed, during extraction, the key factors were the quantity (mass) of the plant material (the final concentration of the extract), choice of solvent, temperature and, of course, extraction technique. Parameters such as density, electrical conductivity, acidity (total acidity and pH), soluble solids content, which are strongly influenced by the temperature system, emphasized the stability and strong solubility of the plant powder of the extracts, especially when individual factors of the extraction methods were studied, such as in ultrasound-assisted extractions. (Many studies on fruit juices are available.) For example, the density of extracts provided information on the materials, their purity, concentration of ingredients, and composition. The density (and concentration) of liquid products had a great influence on their quality, behavior and final use [24,25,26]. Also, the phenomenon of transient cavitation, the main mechanism of ultrasonic action, led to the formation of micro-areas at the level of liquid media characterized by extremely high temperature and pressure, which was manifested at the macro level by a slight increase in system temperature [27].

**Figure 1:**
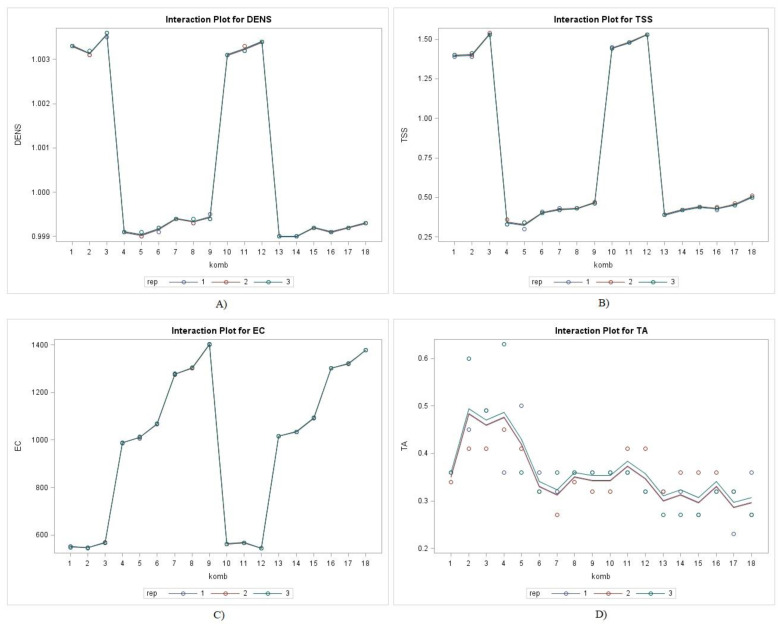
Physicochemical parameters: (**A**) density (g/cm^3^); (**B**) total soluble solids (%); (**C**) electrical conductivity (µm/S); (**D**) total acids (%) of garlic (classic 1–3; UAE 10–12), ginger (classic 4–6; UAE 13–15) and turmeric (classic 7–9; UAE 16–18) extracts.

Ultrasonic technology showed a significant, positive effect on the physicochemical and nutritional properties of various kinds of food, preserving thermolabile ingredients such as vitamins, minerals, phenolics, and pigments. Moreover, it is categorized as a non-thermal method because the temperature is increased only slightly so that it has no significant negative effect on physical and basic chemical parameters like total acid, total soluble solid, color, electrical conductivity, viscosity, and density [28,29]. The analysis of some basic physicochemical properties of the spice extracts is shown in Figure 1.

According to the statistical analysis, all observed physicochemical parameters differed statistically significantly (*p* ≤ 0.0001) between spice type, extraction method and temperature matrix. Also according to the interaction significance analysis (Table 1), it can be concluded that only the interaction among all experimental factors had a significant effect on the observed physicochemical parameters except for SS × ET interaction on pH and SS × T interaction on EC.

Density, electrical conductivity (EC), total soluble solids (TSS), total acids (TA) and the pH of spice extracts differed significantly by spice extract: Garlic had the highest density values (mean 1.0033 g/cm^3^), while ginger had the lowest (mean 0.9991 g/cm^3^); however, ginger had the highest EC values (mean 1330.78 µS/cm), while garlic had the lowest (mean 556.89 µS/cm). Garlic accounted for the highest TSS (mean 1.4%), while ginger showed the lowest (mean 0.39%). The highest TA (mean 0.39%) was in garlic extracts, while the lowest (mean 0.32%) was found in turmeric extracts irrespective of extraction method and matrix temperature. Corresponding to the lower TA value, higher pH values were obtained from the turmeric extracts. As expected, the increase in system temperature had a significant effect on some physicochemical properties of the analyzed spice extracts. Regardless of the species and extraction method, increasing the system temperature (up to 70 °C) had a positive effect on extract density, EC, and TSS, with higher values of the above parameters determined at higher extraction temperatures. UAE had a moderate influence on the analyzed physicochemical properties, since the values of TSS and EC were generally higher in the extracts treated with ultrasound; in contrast, the values of TA and density were lower, emphasizing that the changes recorded were not strongly significant. The results were in agreement with other literature data, as various research studies indicated that ultrasound can influence the increase or decrease in the mentioned parameters [13,15,16]. The main influence of ultrasound manifests itself in transient cavitation, a phenomenon that leads to a slight increase in the temperature of the system. In this study, for extracts treated with dH_2_O at room temperature (21.7 °C), a maximum temperature of 38.6 °C was measured after 30 min of UAE treatment. For samples treated with dH_2_O at 40 °C, the maximum temperature was 53.9 °C, while for samples treated with dH_2_O at 70 °C, the maximum temperature was 79.4 °C. The increase in temperature affects some physical properties of the liquids, but in general the influence of VAE on the physicochemical properties was more evident at higher ultrasound power (amplitude) [23].

**Figure 2:**
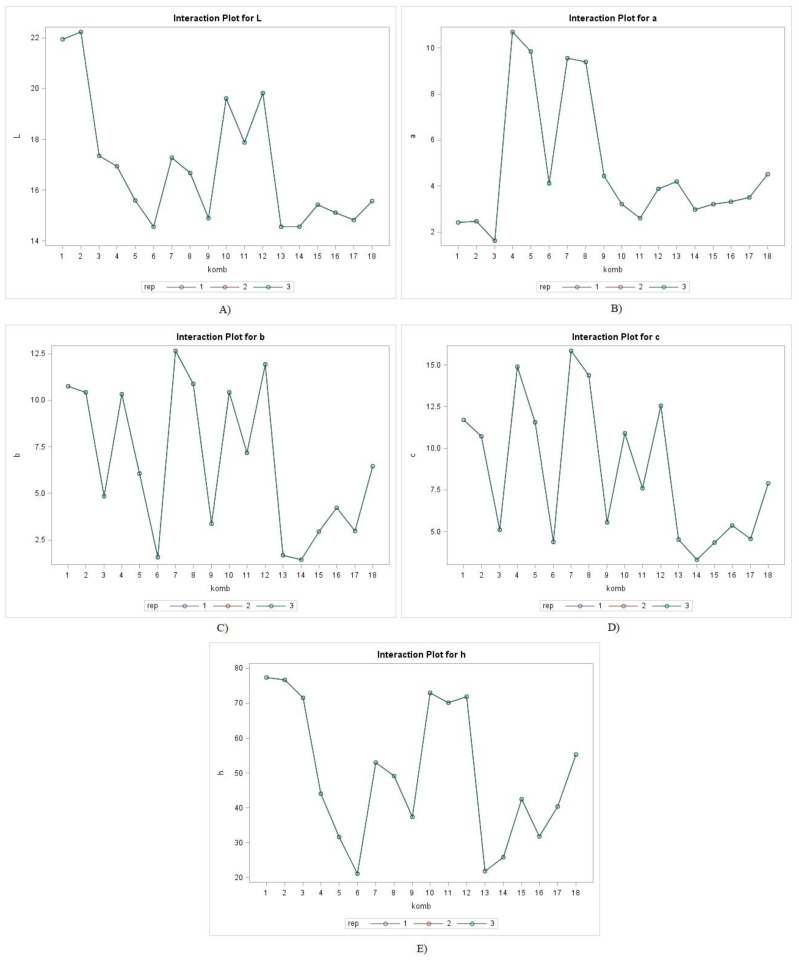
Chromaticity parameters: (**A**) L; (**B**) a; (**C**) b; (**D**) c; (**E**) h of garlic (classic 1–3; UAE 10–12), ginger (classic 4–6; UAE 13–15) and turmeric (classic 7–9; UAE 16–18) extracts.

Product color is one of the characteristics that is particularly important for the consumer because it is also a visual indicator of its quality. During food processing, color parameters are strongly influenced by temperature, light, pH and oxygen. UAE characterized as a non-invasive technique, also has a positive effect on the preservation of organoleptic properties of a product, by being effective in the inactivation of enzymes related to color change [30]. According to the obtained results of color parameters (L, a, b, c, h) presented in Figure 2, it was observed that during the classical extraction with the temperature increase, the samples became darker (L values were lower), while during the UAE treatment, the samples remained lighter, respectively, and the L values of the UAE-treated samples were higher and more characteristic of the color of the spice powder. These results were also confirmed by the values of a, b, c and h, on which the extraction technique showed no significant effect; in contrast, increasing the matrix temperature showed a more significant effect on all observed color parameters. Similar results of UAE effect on color were obtained by different authors [25,31]. One of the effects induced by the transient cavitation in the UAE-treated samples was an oxidation reaction, which occurred as a result of interaction with free radicals and the ultimately affect the color stability [30,32]. According to the interaction significance analysis (Table 2), it can be concluded that the interaction of experimental factors (spice type, extraction technique and temperature) had a significant effect on all observed color parameters, while the interaction SS × ET had a significant effect on the parameters a, b and c, and the interaction ET × T had a significant effect on the parameters L, a, b and c.

### 2.2. Specialized Metabolites and Antioxidant Capacity of Spice Extracts

The results of the content of some specialized metabolites as well as the antioxidant capacity of the prepared spice extracts are presented in Table 3. According to the statistical analysis, significant differences (*p* ≤ 0.0001) were found for all the analysed compounds in the different experimental factors. The spice species differ significantly in vitamin C content, with turmeric standing out as having the highest content. Both temperature and extraction method significantly affected the vitamin C content in all the spice extracts. As expected, the higher matrix temperature (40 and 70 °C) contributed to vitamin C degradation. Thus, its content decreased by 23.1% in garlic samples prepared by classical extraction at 40 °C, 9.9% in ginger and 12.46% in turmeric, while at the higher matrix temperature (70 °C), vitamin C content further decreased by 48.6% in garlic, 20% in ginger and 29.4% in turmeric compared to the matrix room temperature (21.7 °C). Ultrasound-assisted extraction was found to be favourable for vitamin C extraction as significantly higher values were obtained for all spice extracts. The most significant positive UAE effect on vitamin C was observed in ginger extract with 26.6% higher content and in turmeric extract with as much as 38.4% compared to the classically treated samples. Even in UAE-treated samples, the higher matrix temperature (40 and 70 °C) did not cause any degradation in vitamin C content; on the contrary, even higher vitamin C values were found in all spice samples––garlic treated with UAE at 70 °C (ASu3) 30% higher, in ginger (ZOu3) 51% higher, and in turmeric extract (CLu3) 67% higher––compared with the samples treated classically at 70 °C (ASc3, ZOc3, CLc3). Literature data on the effect of UAE on vitamin C content vary as some authors report a positive effect and a higher vitamin C content in UAE-treated samples [25,33,34], while others report a negative effect and a reduction in vitamin C content [32,35]. One possible reason for vitamin C degradation during UAE treatment is the process of sonolysis, i.e., ultrasonic waves splitting or decomposing water molecules to form hydrogen ions (H^+^), free radicals (O^−^, OH^−^, HO^2−^), and hydrogen peroxide (H_2_O_2_). Their interactions, promoted during sonication, caused the occurrence of oxidation that can potentiate vitamin C degradation [36]. Analyzing the interactions of the experimental factors on the vitamin C content (Table 4), it can be observed that the interactions of spice type and extraction technique (SS × ET) and the interaction of spice type and temperature (SS × T) had a significant effect on vitamin C content, while the interaction of all three spice types, extraction technique and temperature (SS × ET × T) had the most significant effect with a determined *p* ≤ 0.0001.

Treatment with ultrasound showed beneficial effects on polyphenolic compounds such as flavonoids, phenolic acids, flavones, flavonols [21,29,37], and the OH radicals that were formed improved the functionality of the mentioned compounds [38]. The results of this study also supported this, because significantly higher levels of total phenols (TPC), flavonoids (TFC) and non-flavonoids (TNFC) were determined in the UAE-treated extracts irrespective of spice type and matrix temperature (Table 3). The highest TPC content (177.32 mg GAE /100 g) was determined in the ginger extract (Zou3) treated with UAE at 70 °C, corresponding to a value about 40% higher compared to the same sample (ZOc3) treated using classical methods. The greatest increase in TPC content, even twofold, was observed in turmeric extract treated with UAE at 70 °C (CLu3) compared to the same sample treated classically (CLc3). The same trend of significant content increase was also observed for TFC (approximately 45% in extract Zou3 compared to ZOc3, and 2 times in extract CLu3 compared to CLc3) and TNFC (37% in extract Zou3 compared to ZOc3; and 2.5 times in extract CLu3 compared to CLc3) during UAE treatment. Comparing the TPC, TFC and TNFC content in extracts of different spice species, it was observed that the highest content was determined in ginger extracts, while the lowest was in garlic for both classical and UAE extraction. The matrix temperature had a significant effect on the TPC, TFC and TNFC content, regardless of the spice type and extraction method. Classical extraction determined significantly higher TPC content in extracts at a matrix temperature of 40 °C compared to classical extraction at room temperature, while a further increase in matrix temperature (70 °C) showed opposite results, respectively: TPC content decreased by 43.5% in garlic extracts, 24.5% in ginger extracts and 26.4% in turmeric extracts compared to the values determined in samples extracted at 40 °C. Increasing the temperature of the system favors the extraction by increasing the solubility of the compounds in the solvent, but the phenolic compounds are susceptible to hydrolysis and oxidation at temperatures higher than 60 °C [39]; therefore, some authors suggest that the extraction temperature of the phenolic compounds should not be higher than 60 °C [40,41]. However, during UAE such a trend concerning the influence of higher matrix temperature on TPC was not observed. In fact, at the higher temperature (70 °C), TPC was as much as 21.6% higher in ginger extract and 15.4% higher in turmeric compared to samples treated with UAE at 40 °C. These results demonstrate the strong effect of the cavitation phenomenon or the ability of ultrasound to break cell walls and cause faster mass transfer, releasing (isolating) cell nutrients (vitamins, minerals, phenols) in solution. The significance of factor interaction (Table 4) shows that the spice type and extraction technique (SS × ET) interaction and the spice type, extraction technique and temperature interaction (SS × ET × T) had a significant effect on TPC, TFC and TNFC content, while for TNFC, the interaction of spice type and temperature (SS × T) also had a significant effect.

Total carotenoid content (TCA) was determined for ginger and turmeric extracts based on their apparent color. The lowest TCA content at room temperature was determined for ginger while the highest was for turmeric. The research results also showed that at higher extraction temperatures (40 and 70 °C), regardless of the extraction method (classical or ultrasonic), the content of TCA increased. Thus, the highest levels for both ginger and turmeric extracts were found at 70 °C. Moreover, ultrasound was found to have had a significantly positive effect on the extraction of TCA; namely, the ginger samples extracted at 70 °C had a TCA content 18.1% higher than the samples classically extracted at the same temperature, while the turmeric extracts even had a TCA content 69.4% higher at the indicated temperature. The positive effect of ultrasonic treatment has also been demonstrated in other studies [29,33]. The significance analysis of the interactions of the experiments (Table 4) showed that the interaction of spice type and extraction technique (SS × ET) and the interaction of spice type, extraction technique and temperature (SS × ET × T) had the strongest influence on the TCA content.

From the spice extracts analyzed, the ABTS assay was used to determine the antioxidant capacity for ginger and turmeric extracts, while this assay was not applicable for garlic. The highest antioxidant capacity from the spice species analyzed was determined for ginger extracts, which was expected due to the highest TPC content determined. The classical treatment at 40 °C increased the antioxidant capacity of turmeric extract by 5.8% compared to the samples extracted at room temperature. This may be attributed to higher TPC content from increased solubility and diffusion coefficient at 40 °C [42]. At 70 °C, the antioxidant capacity of classically treated turmeric extract decreased by 0.66%, which may be attributed to the hydrolysis and oxidation of phenolic compounds at temperatures above 60 °C [39]. Upon ultrasonic treatment at 70 °C, the antioxidant capacity of ginger extract increased by 8.76% and that of turmeric extract by 4% compared to the values determined at 40 °C, which was expected given the TPC values determined for the aforementioned samples. The most significant effect of experimental factor interactions on antioxidant capacity (Table 4) was determined for the interaction of spice type and extraction technique (SS × ET) and for the interaction of spice type, extraction technique and temperature (SS × ET × T).

### 2.3. Antimicrobial Activity of Spice Extracts

#### Growth of Pathogenic Strains

In all inoculated samples, we observed turbidity in the broth medium supplemented with spice extract, which suggests pathogen growth except for inhibited growth of the pathogenic strain *L. monocytogenes* in the media supplemented with the ultrasonic garlic extract.

The colony-forming unit (CFU) value of all pathogenic strains (*Salmonella, L. monocytogenes* and *S. aureus*) used in this study could not be determined as we estimated more than 300 colonies per plate (data not shown).

The added concentration of ginger and turmeric extracts did not significantly reduce the number of selected foodborne pathogens. Similar results for turmeric extracts were observed by Lourenco et al. [43], where they reviewed the antimicrobial effect of turmeric extract on *S. aureus* and the most common pathogen of chicken meat, *Escherichia coli*. Nevertheless, Bader et al. [44] noted the antimicrobial activity of turmeric on pathogenic strains of *S. aureus* and *L. monocytogenes*, but they used a different methodology (minimum inhibitory concentration method) and higher concentrations of extracts than those used in the previous study and ours.

Regarding ginger extract, similar results to ours were noted by Tagoe and Gbadgo [45], but they also used the minimum inhibitory concentration method. They were not able to evaluate the antimicrobial activity of ginger on pathogenic strains of *Salmonella*, *Bacillus cereus*, *Escherichia coli* and *Shigella*. As expected, the authors Nas et al. [46], who used a less concentrated extract of ginger, found an even weaker inhibition of pathogens.

Interestingly, Khazal et al. [47] described the potent antimicrobial activity of ginger extracts against Gram-positive and Gram-negative bacteria, including pathogenic strains of *S. aureus* and *Salmonella*. In addition, ginger extracts were found to lose their antimicrobial activity when exposed to higher extraction temperatures, and antimicrobial efficiency was strongly related to the type of solvent used during extraction. Thus, hexane was found to be the best solvent while distilled water was found to be the worst for the antimicrobial activity potential of the ginger extract [48]. In conclusion, the antimicrobial properties of turmeric and ginger were significantly influenced by the preparation of the extracts and the concentration used.

Garlic extracts obtained after sonication at 21.7 °C and 40 °C showed no significant antimicrobial effect on *Salmonella* and *S. aureus* strains. However, in contrast to turmeric and ginger extract, weaker colony growth of *L. monocytogenes* was observed. Unfortunately, the garlic powder used was not sufficient to produce a higher concentration of extract to inhibit *L. monocytogenes*, so a valid number of colonies could not be determined. Similar results to ours were noted by Yetgin et al. [49]. In their study, they found that garlic extract could suppress the growth of *L. monocytogenes* but had no effect on *Salmonella* and *S. aureus*. The disc diffusion method they used showed that as the concentration of the extract increased, the pathogen inhibitory zone became larger for all three pathogens, not just *L. monocytogenes*.

According to Josipović et al. [13], the main compound responsible for the antimicrobial activity of garlic is the organic component allicin. Because it is heat sensitive and degrades at temperatures above 25 °C, the best method for its extraction is ultrasonication [50]. Complementary to our results also were the findings of Jolly and Menon [51], in which they reported how garlic extracts were able to inhibit the growth of *L. monocytogenes* by increasing the concentration of the extract in the media. Garlic powder extracts also showed their inhibitory potential on several other pathogens belonging to *L. monocytogenes* as reported by other authors [52].

Lastly, we argued that the concentration of the extracts in all tested samples was crucial. Our approach focused more on the dosage ingested by the consumer with food, expecting to notice a slight effect on foodborne pathogens, which we did. The methods of extraction and the temperatures at which they were performed also indicated how difficult it would be to get all the antimicrobial components from the spices into the body. Further studies in this area would be necessary, but quite promising.

## 3. Materials and Methods

### 3.1. Plant Material

For research purposes, the following spices were used: garlic (*Allium sativum* L.), ginger (*Zingiber officinalle* L.) and turmeric (*Curcuma longa* L.). The rhizome powder was purchased at a local specialty herb store. Some additional information about the turmeric product could be gleaned from the existing product label: information on country of origin (India), ecolabel and purity level (98%), while for the ginger powder the country of origin (Pakistan), ecolabel and purity level (96%) were also provided. Garlic powder was cultivated on experimental field of Department of Vegetable Crops in the Faculty of Agriculture at the University of Zagreb. Immediately after harvest, garlic was transported to the Department of Agricultural Technology, Storage and Transport Faculty of Agriculture University of Zagreb where the powder was produced according to following procedure: husks were removed from the garlic cloves, which were then cut into thin slices (1–1.5 mm) and dried by convection in a laboratory drier (INKO ST40, Zagreb, Croatia) at 55 °C till a constant dry matter content of 89.8% was attained. The dried garlic was ground in a laboratory mill (IKA MF-10, Staufen, Germany) to a powdery consistency and passed through a sieve of varying pore size. The approximate size of most particles was 1 mm or smaller, and they were stored in glass containers to further analysis.

### 3.2. Preparation of Spice Extracts

Spices powders, each 4.0000 ± 0.0001 g, were weighed in laboratory glass flasks and a total volume of 250 mL distilled water at three different temperatures (21.7, 40 and 70 °C) was added. For the purpose of classical extraction, the spice samples were allowed to stand for 30 min with intermittent agitation and maintain the temperature of the system. For the purposes of the UAE, the prepared spices samples (4.0000 ± 0.0001 g) received 250 mL dH_2_O and were immediately placed into an ultrasonic bath (Bandelin RK 103H, Berlin, Germany) with a nominal power of 140 W and frequency of 35 kHz. UAE was performed for 30 min while temperatures of 40 and 70 °C were maintained in the bath. Throughout the process, the temperature of the samples was measured with an infrared thermometer (Uni-trend technology, UT 3000C, Dongguan, Guangdong Province, China) every 30 s. In both the classic and UAE extraction, the laboratory flasks were sealed with silicone lids to prevent loss of extract volume at the higher evaporating temperatures. The final volume of each prepared extract was checked and averaged 250 mL. After 30 min, the samples were filtered and prepared for further chemical analysis. The whole design of the experiment is shown in Table 5.

### 3.3. Determination of Physicochemical Properties of Spice Extracts

The following physicochemical parameters of the spice extracts were determined: density (g/cm^3^) by digital densitometer (Mettler Toledo, Densito 30PX, Switzerland), electrical conductivity (µS/cm) by conduct meter (Mettler Toledo, SevenEasy Conductivity, Switzerland), total soluble solids (TSS, %) by digital refractometer (Mettler Toledo, Refracto 30PX, Switzerland), total acid content (TA, %) according to standard method of potentiometric titration [53], pH value by digital pH meter (Mettler Toledo, SevenMulti, Greifensee, Switzerland) and color by colorimeter (ColorTec PCM+, Meschede, Germany) according to the CIELAB method.

### 3.4. Determination of Specialized Metabolites Content and Antioxidant Capacity of Spice Extracts

Vitamin C (mg/100 g) was determined by titration with 2,6-dichlorophenolindophenol according to the method previously described by AOAC [54]. Vitamin C was isolated by homogenizing 10 g ± 0.01 of plant extract with 100 mL of 2% (v/v) oxalic acid. The prepared solution was filtered through Whatman filter paper, and the filtrate was obtained in a volume of 10 mL. This was then titrated with freshly prepared 2,6-dichloroindophenol until the appearance of a pink coloration. Moreover, total polyphenols, flavonoids and non-flavonoids (mg GAE/100 g) were evaluated spectrophotometrically (UV-1650 PC, Shimadzu, Kyoto, Japan) by measuring their color intensity from a reaction with Folin–Ciocalteu reagent at a wavelength of 750 nm, distilled water as a blank probe, and gallic acid as an external standard for analysis of total phenolics and catechin for analysis of total flavonoids according to method described by Shukla et al. [55] with some minor modification. One milliliter of the prepared spice extracts and 1 mL of the reagent Folin–Ciocalteu (diluted with water in a ratio of 1:2) were placed into a 50 mL volumetric flask. The prepared solution was allowed to stand at room temperature for 3 min. After 3 min, 3 mL of saturated sodium carbonate solution was added and the flask was filled to the mark with distilled water. The prepared samples were allowed to stand at room temperature for 3 h with occasional shaking. After 3 h, measurements of the phenolic compounds were carried out; Total carotenoids (mg/g) were measured spectrophotometrically using the method described by Holm [56] and Wettstein [57] at a wavelength of 440 nm and acetone (p.a.) as a blank probe. The carotenoids of the prepared extracts were dissolved in a total volume of 15 mL of acetone (p.a.) and passed through a sodium sulfate layer to remove excess water. The resulting carotenoid eluate was dissolved in a known volume and the absorbance read off spectrophotometrically. To obtain the results of pigment content based on complained absorbance, the Holm–Wettstein equation was used (1). The final results for total carotenoid content was expressed in mg/g.

Total carotenoids =4.695 × A_440 − 0.268 × (total chlorophylls) [mg/L](1)

Antioxidant capacity of spice extracts was determined spectrophotometrically using the ABTS radical cation (2, 2′-azinobis (3-ethylbenzothiazoline-6-sulfonic acid)) and ethanol (96%) as a blank probe at a wavelength of 734 nm by method described by Re et al. [58]. Trolox (6-hydroxy-2,5,7,8-tetramethylchroman-2-carboxylic acid, Sigma-Aldrich, St. Louis, MO, USA) was used as the external standard. Trolox was prepared as a stock standard and appropriate dilutions for the preparation of the calibration curve were also prepared. To obtain an ABTS radical (ABTS-1), 5 mL of ABTS solution (7 mM) and 88 mL of potassium persulfate solution (140 mM) were mixed and allowed to stand for 16 h in the dark at room temperature. On the day of analysis, a 1% ABTS-1 solution (in 96% ethanol) was prepared. A total of 160 µL of the spice extract was injected directly into the cuvette and mixed with 2 mL of stable ABTS-1. The final results were expressed as mmol TE/L.

### 3.5. Determination of Antimicrobial Activity of Spice Extracts

The antimicrobial activity was performed using a previously described method by Kim et al. [59] with modifications. All media used were purchased from Biolife (Milan, Italy). Briefly, the food pathogen strains belonging to *Salmonella sp*., *Listeria monocytogenes* and *Staphylococcus aureus* were obtained from University hospital “Sveti Duh”, Zagreb, Croatia. The strains used were characterized and tested for purity by Gram stain and biochemical tests, API 20E (bioMerieux, Hazelwood, MO, USA) for *Salmonella sp.* and BBL Crystal kit (Difco, Becton Dickinson, Sparks, MD, USA) for *Listeria monocytogenes* and *Staphylococcus aureus*. All strains were handled in the same way and were grown individually for 18 to 24 h at 30 °C in 5 mL of rich broth BHI media. Five hundred microliters of the late log-phased overnight culture (giving a microbial load of approximately 4.0 to 6.0 CFU mL^−1^) was added to freshly prepared 5 mL BHI media supplemented with a spice concentration of 0.016 g/mL and conditions of the spice extracts (garlic, ginger and turmeric, respectively). Each spice extract condition was monitored in triplicate for each strain. One hundred microliters were applied on to BHI dishes and the number of viable colonies was determined post-incubation at 30 °C for 48 h in aerobic conditions.

Control plates were grown under the same conditions. For the positive control we grew the food pathogen strains in rich broth media without spice extracts so that we could appreciate the full pathogen growth; for the negative control we only plated the broth BHI media containing the spice extracts with no pathogen strains to exclude possible internal contamination.

The antimicrobial activity and potential suppression of foodborne pathogens was established by comparison of the growth obtained in the control plates to those grown on the spice extract plates.

### 3.6. Statistical Analysis

The data obtained were analyzed using SAS^®^ version 9.3 [60]. Treatments were arranged in a randomized complete block design with three replicates. A generalized linear model (PROC GLM) with replicates and interactions of spice species (garlic, ginger, turmeric), extraction technique (classical, ultrasound-assisted) and temperature (21.7, 40 and 70 °C) was used for analysis. Mean values were compared using t-test (Least Significant Difference, LSD) and considered significantly different at *p* ≤ 0.01.

## 4. Conclusions

According to the obtained results, it can be concluded that garlic, ginger and turmeric extracts are a rich source of specialized metabolites, i.e., bioactive compounds. Turmeric extract was found to contain the highest amount of vitamin C, while ginger had the highest levels of TPC, TFC, and TFNC regardless of treatment technique. Moreover, the treatment method and application of higher matrix temperatures affected the basic physicochemical parameters. The values of density and electrical conductivity of the extracts increased with the increase in system temperature irrespective of the treatment method. Also, the increase in system temperature as well as the application of ultrasound affected the increase in TSS content. Ultrasonic treatment had a significant effect on the color of the spice extracts: at higher temperatures it favored the increase in the chromaticity parameter b value of turmeric and ginger extracts, indicating a higher content of yellow color, which was predominantly due to curcuminoids in the mentioned species. Ultrasound treatment had a positive effect on all the specialized metabolites analyzed––Vitamin C, total phenols, flavonoids, non-flavonoids and β-carotene––and on the antioxidant capacity of all spice extracts. All the different concentrations of spice extracts were not sufficient for complete inhibition of the pathogenic bacterial strains included in this study; however, only the garlic extracts affected the growth and number of colonies of *L. monocytogenes*. The most important thing to emphasize is that ultrasound slowed the growth of pathogenic bacteria colonies, so further research is needed to determine the influence of ultrasound treatment on antioxidant components and antimicrobial activity.

## Figures and Tables

**Table 1:** Significance analysis of interactions of experiment factors for analysed physicochemical parameters.

Interactions	Density	TSS	EC	TA	pH
	Pr > F	Pr > F	Pr > F	Pr > F	Pr > F
SS × ET	0.1424	0.3778	0.5696	0.2018	0.0160
SS × T	0.0697	0.1059	≤0.0001	0.1872	0.1906
ET × T	0.9916	0.9923	0.9891	0.7009	0.6942
SS × ET × T	≤0.0001	≤0.0001	≤0.0001	0.0480	≤0.0001

SS × ET, interaction between spice species and extraction technique; SS × T, interaction between spice species and temperature; ET × T, interaction between extraction technique and temperature; SS × ET × T, interaction among spice species, extraction technique and temperature.

**Table 2:** Significance analysis of interactions of experiment factors for chromaticity parameters.

Interactions	L	a	b	c	h
	Pr > F	Pr > F	Pr > F	Pr > F	Pr > F
SS × ET	0.7951	≤0.0001	0.0085	0.0014	0.9417
SS × T	0.5841	0.3869	0.9641	0.8755	0.7618
ET × T	0.0250	0.0045	≤0.0001	≤0.0001	0.0794
SS × ET × T	≤0.0001	≤0.0001	≤0.0001	≤0.0001	≤0.0001

SS × ET, interaction spice species and extraction technique; SS × T, interaction spice species and temperature; ET × T, interaction extraction technique and temperature; SS × ET × T, interaction spice species, extraction technique, and temperature.

**Table 3:** Specialized metabolites content, antioxidant capacity of spice extracts.

Sample ID	VIT_C	TPC	TFC	TNFC	TCA	ANT_CAP
	*p* ≤ 0.0001	*p* ≤ 0.0001	*p* ≤ 0.0001	*p* ≤ 0.0001	*p* ≤ 0.0001	*p* ≤ 0.0001
**Classic extraction**						
ASc1	8.61 ^ef^ ± 0.68	43.67 ^l^ ± 1.24	10.54 ^k^ ± 1.64	33.11 ^i^ ± 0.6	ND	ND
ASc2	6.62 ^g^ ± 0.01	41.67 ^lm^ ± 1.17	6.45 ^l^ ± 0.96	35.24 ^h^ ± 0.8	ND	ND
ASc3	4.42 ^i^ ± 0.33	23.52 ^o^ ± 1.91	1.84 ^n^ ± 0.56	23.19 ^l^ ± 0.42	ND	ND
ZOc1	6.62 ^g^ ± 0.01	146.02 ^c^ ± 1.15	94.58 ^b^ ± 0.98	51.44 ^d^ ± 0.96	0.57 ^j^ ± 0.01	2.30 ^a^ ± 0.87
ZOc2	5.29 ^hi^ ± 0.88	166.80 ^b^ ± 0.50	110.58 ^a^ ± 1.07	56.16 ^c^ ± 0.51	0.71 ^i^ ± 0.01	2.27 ^a^ ± 0.52
ZOc3	5.96 ^gh^ ± 0.01	125.86 ^e^ ± 0.60	63.84 ^ef^ ± 1.01	62.02 ^b^ ± 0.65	0.91 ^h^ ± 0.1	2.28 ^a^ ± 0.14
CLc1	10.81 ^d^ ± 0.77	67.57 ^j^ ± 0.50	48.81 ^i^ ± 1.11	23.76 ^l^ ± 0.61	1.1 ^g^ ± 0.3	2.17 ^cd^ ± 0.04
CLc2	12.35 ^c^ ± 0.38	77.86 ^i^ ± 0.64	49.54 ^h^ ± 0.43	28.32 ^k^ ± 0.83	1.73 ^d^ ± 0.1	2.30 ^a^ ± 0.27
CLc3	8.84 ^ef^ ± 0.33	57.24 ^k^ ± 0.40	32.88 ^j^ ± 0.15	24.37 ^l^ ± 0.28	4.17 ^c^ ± 0.1	2.29 ^a^ ± 0.67
**Ultrasonic-Assisted Extraction**						
ASu1	8.26 ^ef^ ± 0.33	39.12 ^m^ ± 0.74	5.32 ^lm^ ± 0.68	33.61 ^hi^ ± 0.48	ND	ND
ASu2	8.07 ^f^ ± 0.01	34.48 ^n^ ± 0.80	3.41 ^mn^ ± 1.04	30.95 ^j^ ± 0.62	ND	ND
ASu3	5.76 ^gh^ ± 0.01	23.65 ^o^ ± 0.99	4.87 l^m^ ± 1.06	18.79 ^m^ ± 0.16	ND	ND
ZOu1	6.53 ^g^ ± 0.33	115.51 ^f^ ± 0.45	66.57 ^de^ ± 0.46	48.94 ^e^ ± 0.56	1.15 ^f^ ± 0.01	2.10 ^d^ ± 0.69
ZOu2	8.84 ^ef^ ± 0.33	138.93 ^d^ ± 1.96	75.82 ^c^ ± 1.99	63.10 ^b^ ± 0.11	1.23 ^e^ ± 0.02	1.98 ^e^ ± 0.12
ZOu3	9.03 ^e^ ± 0.67	177.32 ^a^ ± 0.67	92.53 ^b^ ± 0.61	84.88 ^a^ ± 1.14	1.15 ^f^ ± 0.06	2.18 ^bc^ ± 0.27
CLu1	14.41 ^b^ ± 0.33	97.93 ^h^ ±1.15	55.26 ^g^ ± 1.64	42.66 ^g^ ± 0.77	11.53 ^b^ ± 0.1	2.25 ^ab^ ± 0.87
CLu2	17.10 ^a^ ± 0.33	108.79 ^g^ ± 0.43	62.07 ^f^ ± 0.47	46,73 ^f^ ± 0,79	13.45 ^a^ ± 0.1	2.09 ^d^ ± 0.42
CLu3	14.79 ^b^ ± 0.58	128.67 ^e^ ± 1.11	67.17 ^d^ ± 0.22	63.50 ^b^ ± 2.32	13.45 ^a^ ± 0.1	2.18 ^bc^ ± 0.03

VIT_C, vitamin C content (mg/100 g); TPC, total phenol content (mgGAE/100 g); TFC, total flavonoid content (mgCAT/100 g); TNFC, total non-flavonoid content (mgGAE/100 g); TCA, total carotenoid content (µg/g); ANT_CAP, antioxidant capacity (mmol TE/L); ND, not determined. Different letters (a–o) indicate significant differences between means at *p* ≤ 0.0001.

**Table 4:** Significance analysis of interactions of experiment factors for specialized metabolites.

Interactions	VIT_C	TPC	TFC	TNFC	TCA	ANT_CAP
	Pr > F	Pr > F	Pr > F	Pr > F	Pr > F	Pr > F
SS × ET	0.0003	≤0.0001	0.0002	≤0.0001	≤0.0001	≤0.0001
SS × T	0.0286	0.1924	0.5473	0.0012	0.9039	0.6133
ET × T	0.4959	0.3617	0.3613	0.4410	0.9548	0.9858
SS × ET × T	≤0.0001	≤0.0001	≤0.0001	≤0.0001	≤0.0001	≤0.0001

SS × ET, interaction of spice species and extraction technique; SS × T, interaction of spice species and temperature; ET × T, interaction of extraction technique and temperature; SS × ET × T, interaction of spice species, extraction technique and temperature.

**Table 5:** Experimental conditions of classic (CLAS) and ultrasound-assisted extraction (UAE).

Extraction Method	Spice Species	Solvent and Volume (mL)	Time (min)	Temperature (°C)	Ultrasonic Bath	Sample ID
CLAS	Garlic	dH_2_O, 250	30	21.7	-	ASc1
CLAS	Garlic	dH_2_O, 250	30	40	-	ASc2
CLAS	Garlic	dH_2_O, 250	30	70	-	ASc3
CLAS	Ginger	dH_2_O, 250	30	21.7	-	ZOc1
CLAS	Ginger	dH_2_O, 250	30	40	-	ZOc2
CLAS	Ginger	dH_2_O, 250	30	70	-	ZOc3
CLAS	Turmeric	dH_2_O, 250	30	21.7	-	CLc1
CLAS	Turmeric	dH_2_O, 250	30	40	-	CLc2
CLAS	Turmeric	dH_2_O, 250	30	70	-	CLc3
UAE	Garlic	dH_2_O, 250	30	21.7	35 kHz 140 W	ASu1
UAE	Garlic	dH_2_O, 250	30	40	35 kHz 140 W	ASu2
UAE	Garlic	dH_2_O, 250	30	70	35 kHz 140 W	ASu3
UAE	Ginger	dH_2_O, 250	30	21.7	35 kHz 140 W	ZOu1
UAE	Ginger	dH_2_O, 250	30	40	35 kHz 140 W	ZOu2
UAE	Ginger	dH_2_O, 250	30	70	35 kHz 140 W	ZOu3
UAE	Turmeric	dH_2_O, 250	30	21.7	35 kHz 140 W	CLu1
UAE	Turmeric	dH_2_O, 250	30	40	35 kHz 140 W	CLu2
UAE	Turmeric	dH_2_O, 250	30	70	35 kHz 140 W	CLu3

## Data Availability

Not Applicable.

## Sample Availability

Samples of the compounds are available from the authors.
